# Determining the optimal PD‐1/PD‐L1 inhibitors for the first‐line treatment of non‐small‐cell lung cancer with high‐level PD‐L1 expression in China

**DOI:** 10.1002/cam4.4191

**Published:** 2021-08-12

**Authors:** Meng‐Meng Teng, Si‐Ying Chen, Bo Yang, Yan Wang, Rui‐Ying Han, Meng‐Na An, Ya‐lin Dong, Hai‐Sheng You

**Affiliations:** ^1^ Department of Pharmacy The First Affiliated Hospital of Xi'an Jiaotong University Xi'an China; ^2^ Department of Pharmacy The Second Affiliated Hospital of Xi'an Jiaotong University Xi'an China

**Keywords:** cost‐effectiveness, efficacy, non‐small lung cancer (NSCLC), PD‐1/PD‐L1 inhibitors, safety

## Abstract

**Background and Objective:**

The programmed death 1 and ligand (PD‐1/PD‐L1) inhibitors have significantly altered therapeutic perspectives on non‐small‐cell lung cancer (NSCLC). However, their efficacy and safety are unknown since direct clinical trials have not yet been performed on them. It is also necessary to determine the economics of PD‐1/PD‐L1 inhibitors due to their high cost. The aim was to evaluate the efficacy, safety, and cost‐effectiveness of PD‐1/PD‐L1 inhibitor monotherapy for advanced NSCLC patients in China with high PD‐L1 expression as first‐line treatment.

**Methods:**

From the PubMed, Cochrane, and Web of Science databases, we retrieved survival, progression, and safety data on PD‐1/PD‐L1 inhibitor monotherapy for advanced NSCLC patients. A network meta‐analysis (NMA) was performed to consider PD‐1/PD‐L1 inhibitors in efficacy and safety. A Markov model with a full‐lifetime horizon was adopted. Clinical and utility data were collected through the trial. The cost per quality‐adjusted life year (QALY) was as incremental cost‐effectiveness ratio (ICER). Sensitivity analyses were performed.

**Results:**

This study included five phase III clinical trials using four drugs: nivolumab, pembrolizumab, atezolizumab, and durvalumab. The NMA demonstrated that the four drugs had similar efficacy and safety, while pembrolizumab and atezolizumab were better for than for nivolumab (hazard ratio (HR) = 0.66, 95% confidence intervals (CIs): 0.46–0.95 and HR = 0.59, 95%CI: 0.37–0.94) in progression‐free survival (PFS), and the risk of a severe adverse event was higher for atezolizumab than for nivolumab and pembrolizumab. Compared with nivolumab, durvalumab, pembrolizumab, and atezolizumab had QALY of 0.19, 0.38, and 0.53, respectively, which induced ICERs of $ 197,028.8/QALY, $ 111,859.0/QALY, and $ 76,182.3/QALY, respectively.

**Conclusion:**

The efficacy and safety are similar among types of PD‐1/PD‐L1‐inhibitor monotherapy. The cost‐effectiveness of nivolumab appears optimal, but the other PD‐1/PD‐L1 inhibitors are not as cost‐effective for the first‐line treatment of advanced NSCLC in China.

## Graphical abstract

A network meta‐analysis was performed to indirectly consider the efficacy and safety of PD‐1/PD‐L1 inhibitors, and the Markov model was established to compare their economic.The efficacy and safety of the four PD‐1/PD‐L1 inhibitors analyzed in this study are similar.From the perspective of the Chinese healthcare system, nivolumab therapy is a cost‐effective alternative to other drugs as first‐line treatments for advanced NSCLC with high‐level PD‐L1 expression.

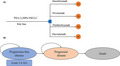

## 1 INTRODUCTION

Lung cancer is the most common malignant cancer in the world, as well as the most common cause of cancer‐related deaths.^1^ Non‐small cell lung cancer (NSCLC) accounts for more than 80% of all lung cancers, and most NSCLC cases are locally advanced or metastatic at the time of diagnosis.^2^ Platinum‐based chemotherapy is often recommended for advanced or metastatic NSCLC patients but has a survival rate lower than 20%,^3^, ^4^ and therefore does not significantly prolong overall survival (OS). However, immunotherapy has shown enormous potential to further improve the prognosis for lung cancer patients, especially the programmed death 1 and ligand (PD‐1/PD‐L1) inhibitors.^5^, ^6^, ^7^

The PD‐1/PD‐L1 inhibitors have rapidly received Food and Drug Administration agreement due to their strong clinical efficacy, longer survival, and less severe side effects.^8^, ^9^, ^10^, ^11^ PD‐1/PD‐L1 inhibitors in several three‐phase clinical trials prolonged survival times and decreased adverse events during NSCLC treatment, especially when patients had a high PD‐L1 expression (≥50%).^9^, ^10^, ^11^, ^12^, ^13^ These results have greatly altered the conventional management of advanced or metastatic NSCLC. However, there is an absence of results directly comparing several different PD‐1/PD‐L1 inhibitors. Nevertheless, the cost of these breakthrough treatments must be considered.^14^, ^15^ The rational use of PD‐1/PD‐L1 inhibitors is urgent, but this remains to be determined.

Ensuring appropriate and sustainable use of targeted treatments for analyzing the efficacy, safety, and cost‐effectiveness of PD‐1/PD‐L1 inhibitors on NSCLC is vital.^14^, ^16^, ^17^, ^18^ This study investigated the efficacy, safety, and cost‐effectiveness of PD‐1/PD‐L1 inhibitors as a first‐line treatment for advanced or metastatic NSCLC with high‐level PD‐L1 expression.

## 2 METHODS

### 2.1 Network meta‐analysis

Direct clinical trials are insufficient for PD‐1/PD‐L1‐inhibitor monotherapy as a first‐line treatment for advanced or metastatic NSCLC with high‐level PD‐L1 expression. A network meta‐analysis (NMA) was therefore performed to evaluate the different PD‐1/PD‐L1 inhibitors in efficacy and safety. Two authors conducted independent reviews of PubMed, Cochrane Library, and Web of Science, published before January 1, 2021, using the search terms shown in supplement Table S1. The search term included “NSCLC,” “anti‐PD‐1,” “anti‐PD‐L1,” “first line,” “randomized controlled trial,” and so on. This analysis included randomized controlled trials with NSCLC eligible for first‐line treatment. Eligible studies included patients with high‐level PD‐1 expression (≥50%), and PD‐1/PD‐L1 inhibitors monotherapy. Eligible studies were also required to report at least an assessment of survival (OS and PFS) and safety. The analysis excluded studies including patients with<18 years old, incomplete or duplicate data, and treatment which included PD‐1/PD‐L1 inhibitor combination with others drugs. The Cochrane risk of bias tool for randomized controlled trials was used to assess the quality and risk of bias of studies included in the analysis. The NMA was performed using graph theory implemented by the netmeta R package.

### 2.2 Analytical overview and model structure

**FIGURE 1:**
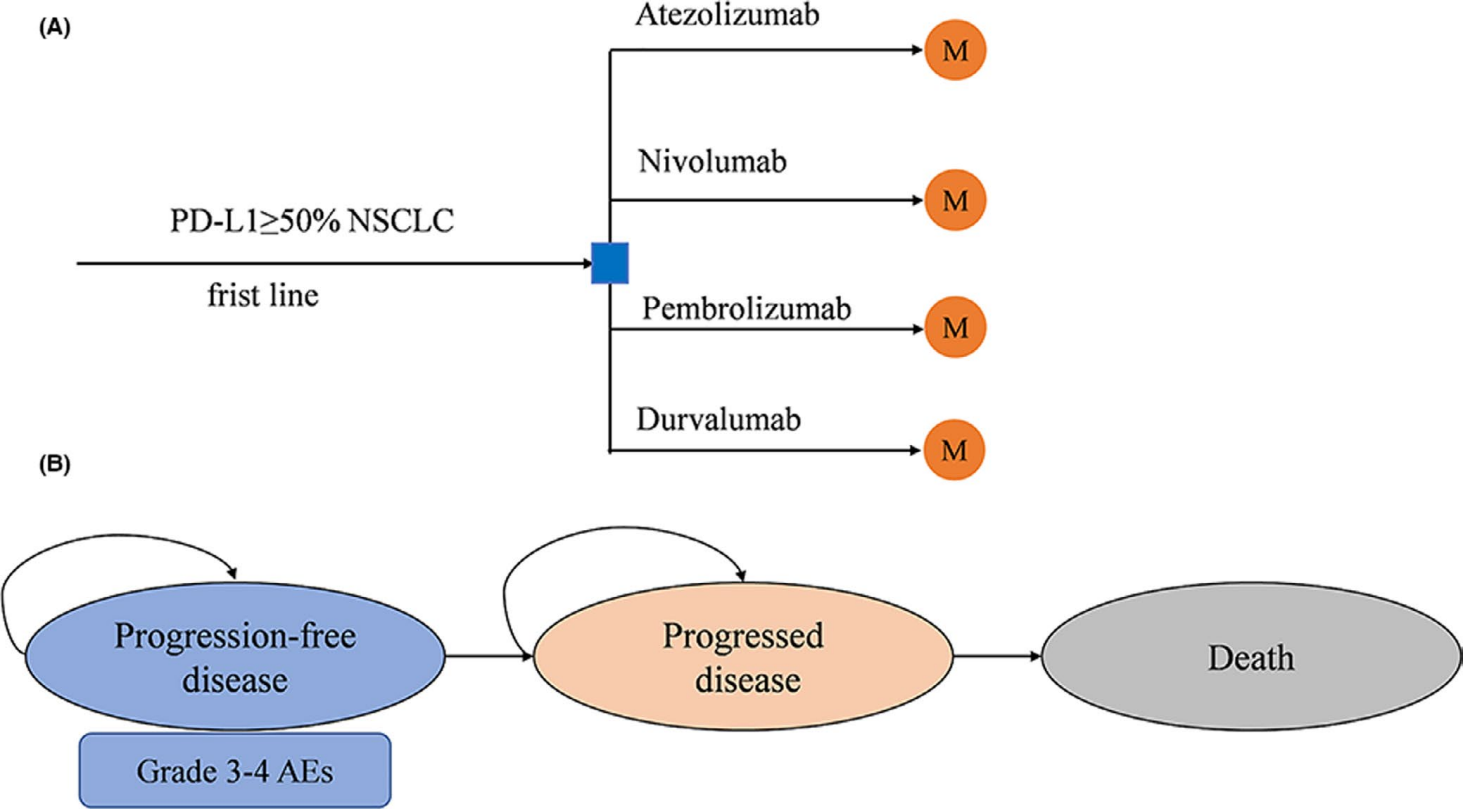
Model structure. (A) The framework of the decision tree; (B) Markov model. PD‐L1, programmed death‐ligand 1; NSCLC, non‐small lung cancer; M, Markov node; AE, adverse events

We constructed a Markov model to determine the clinical and economic outcomes of PD‐1/PD‐L1 inhibitors as first‐line treatments for advanced NSCLC, since this approach is effective for analyzing individual patient‐level data. Virtual patient‐level data were reconstructed. A hypothetical cohort about advanced NSCLC with high‐level PD‐L1 expression was constructed to compare the four potential competing targeted treatment drugs of nivolumab, pembrolizumab, atezolizumab, and durvalumab (Figure 1A). Health and economic outcomes were determined using a Markov model process which considered the three exclusive health states including progression‐free disease (PFD), progressed disease (PD), and death. The Markov process was 21 days and PFD was the initial health state of all patients. The model was run until 99% of patients entered the death state with a lifetime horizon. The risk of disease progression or death was determined by PFS and OS data from the NMA and previously published trials.

The primary outcomes were quality‐adjusted life year (QALY) and cost. Costs and QALY were reduced by 5% annually in line with Chinese guidelines for pharmacoeconomic evaluations.^19^ All costs are presented in 2020 US dollars. Incremental cost‐effectiveness ratios (ICERs) were examined and presented as cost per QALY gained. We followed recommendations for the cost‐effectiveness threshold of three times the per capita gross domestic product of China in 2020. We used three times the per capita gross domestic product of China in 2020 ($32,517.0) as the cost‐effectiveness threshold according to the guideline recommendations.^19^

### 2.3 Clinical data

We indirectly compared the survival rates of the four drugs. WebPlotDigitizer was used to construct Kaplan–Meier curves based on clinical data to project outcomes, and R package was used for fully parametric modeling of the survival data. Weibull survival models were fitted to the Kaplan–Meier survival curves for atezolizumab from IMpower 110 trial,^12^ which demonstrated the best fit for the Kaplan–Meier survival data. Table 1 lists the parameters of the estimated Weibull scale (λ) and shape (γ). The survival probability at time (t) was calculated using the following formula: *S*(*t*) = *P*_(_ *_T_*_≥_ *_t_*_)_ = exp(−λ*t* ^γ^). The transition probability from PFD to PS at a given cycle *t* was calculated by using the Weibull scale (λ) and shape (γ). The other three drugs were derived using the adjusted Weibull scale and shape parameters. The hazard ratios (HRs) of PFS and OS were generated using NMA for these drugs relative to the atezolizumab strategy considered in the economic model.

**TABLE 1:** Cost and utility data

Parameters	Base	Range	Distribution	Reference
Cost (US $)				
Pembrolizumab per cycle	5386.7	–	Fixed in PSA	Local
Atezolizumab per cycle	4930.3	–	Fixed in PSA	Local
Nivolumab per cycle	4171.2	–	Fixed in PSA	Local
Durvalumab per cycle	6117.5	–	Fixed in PSA	Local
Inpatient cost per cycle	55.6	41.7–69.4	Lognormal	^31^
Progression disease treatment per cycle	854.1	706.5–992.4	Lognormal	^32^
Best supportive care per cycle	337.5	158.7–793.7	Lognormal	^31^
Terminal treatment per cycle	2627.8	2291.8–2966.6	Lognormal	^32^
AEs managing cost per cycle	362.2	271.6–452.7	Lognormal	^32^
Severe adverse events rate (%)				
Nivolumab	17.60	–	Beta	^9^
Pembrolizumab	19.49	–	Beta	11, 33
Atezolizumab	30.07	–	Beta	^12^
Durvalumab	14.90	–	Beta	^13^
Utility of health states per event				
PFS	0.804	0.643–0.965	Beta	^34^
PS	0.321	0.257–0.385	Beta	^34^
Discount rate (%)	5	0–8	Fixed in PSA	^19^
Weibull distribution parameters				
Atezolizumab, OS, scale (Weibull), λ	0.055227	–	Fixed in PSA	^12^
Atezolizumab, OS, shape (Weibull), γ	0.724424	–	Fixed in PSA	^12^
Atezolizumab, PFS, scale (Weibull), λ	0.119257	–	Fixed in PSA	^12^
Atezolizumab, PFS, shape (Weibull), γ	0.701234	–	Fixed in PSA	^12^

Abbreviations: HR: Hazard ratio; NMA: Network meta‐analysis; OS: Overall survival; PFS: Progression‐free survival; PS: Progression survival; PSA: Probabilistic sensitivity analysis.

### 2.4 Cost and utility data

Analyses were conducted from the perspective of the Chinese healthcare system. The direct medical costs were considered in the model, which included the drug costs, concomitant medication during therapy, management of severe adverse events (SAEs), routine follow‐ups, and terminal care (Table 1). All costs were adjusted to 2020 US dollars using the local Consumer Price Index (1 US dollar = CNY ¥ 6.5).

### 2.5 Sensitivity analyses

We conducted the univariate and probabilistic sensitivity analyses. One‐way sensitivity analysis tested the variance in potential parameter values of the models (Table 1). Probabilistic sensitivity analysis (PSA) was analyzed using a Monte Carlo simulation, which incorporated the probability distribution including natural history parameters, HRs, costs, and utilities. Standard methods were adopted for defining uncertainty among parameters. The beta distribution was performed to the transition probability, proportion, and utility parameters, and the log‐normal distribution to HRs parameters and costs. This study applied 10,000 replications to obtain a series of 10,000 outcome estimates. Cost‐effectiveness acceptability curves (CEAC) presented the probability of a treatment being cost‐effective against willingness‐to‐pay values.

## 3 RESULT

### 3.1 Network meta‐analysis

**FIGURE 2:**
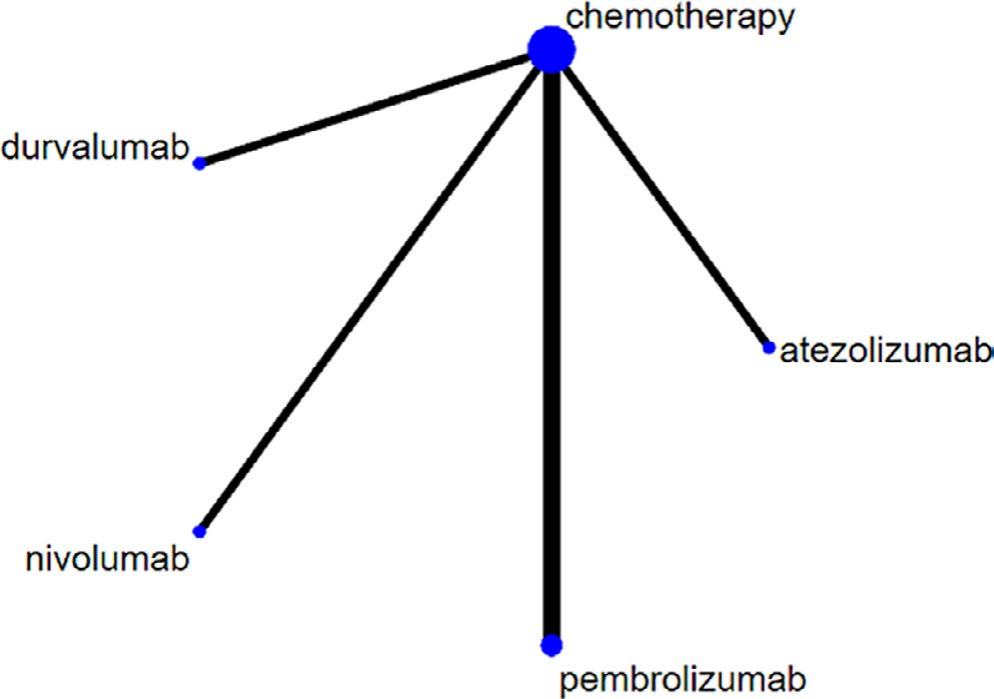
Network meta‐analysis

**FIGURE 3:**
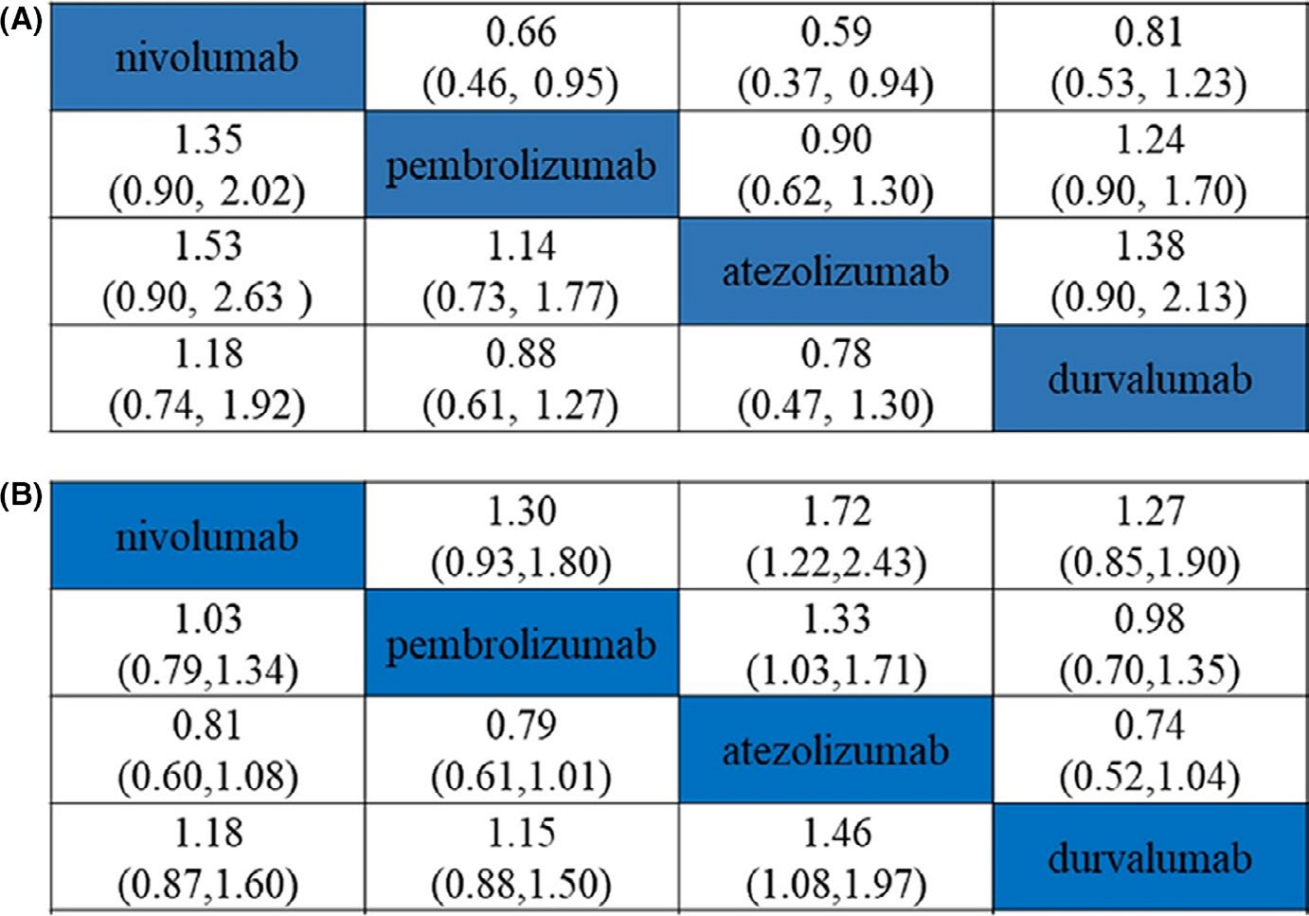
The network meta‐analysis of efficacy and safety. (A) OS at the bottom left, PFS at the top right; (B) Adverse reactions at the bottom left, and serious adverse reactions at the top right

Because of the non‐availability of direct head‐to‐head clinical trial data, an indirect comparison approach was conducted to evaluate the efficacy of PD‐1/PD‐L1 inhibitors as first‐line treatments for advanced NSCLC with high‐level PD‐L1 expression. In total, 650 articles were screened from searches of the databases, after removing duplicates, screening of abstracts, and full‐text article assessed, five articles met the full inclusion criteria (Supplementary Figure S1). The five clinical studies were the KEYNOTE‐024, KEYNOTE‐042, CheckMate‐026, IMpower 110, and MYSTIC,^9^, ^11^, ^12^, ^13^, ^20^ for constructing the network, which all involved nivolumab, pembrolizumab, atezolizumab, or durvalumab. Study characteristics are summarized in Supplementary Table S2. In brief, all studies selected for inclusion were randomized controlled studies, three of the studies used double‐blinding and two was open‐label. Overall, the quality of the included studies was considered relatively low (Supplementary Figure S2). After HRs of OS, PFS, and adverse event data were extracted from these studies, the NMA was conducted based on a fixed‐effects model to consider heterogeneity (*I* ^2^ = 13%) (Figure 2). Figure 3 shows the HRs of OS and PFS, and risk rates (RR) of adverse events and SAE. PFS was marginally better for pembrolizumab and atezolizumab than for nivolumab (HR = 0.66, 95% confidence intervals (CI): 0.46–0.95) and HR = 0.59, 95%CI: 0.37–0.94), whereas OS did not differ significantly between these two interventions (Figure 3A). Only atezolizumab had significant higher SAE safety outcomes than pembrolizumab (RR = 0.66, 95%CI: 0.46–0.95) and nivolumab (RR = 1.72, 95%CI: 1.22–2.43) (Figure 3B).

### 3.2 Base‐case analysis

Compared with nivolumab, durvalumab, pembrolizumab, and atezolizumab treatment strategies increased QALY by 0.19, 0.38, and 0.53, respectively, with incremental costs of $ 37,425.6, $ 42,108.1, and $ 39,758.9, which induced ICERs of $ 197,028.8/QALY, $ 111,859.0/QALY, and $ 76,182.3/QALY, respectively (Table 2). These results indicated that atezolizumab and pembrolizumab had better cost‐effectiveness than durvalumab ($25,108.6 and $7,029.2 per QALY), and atezolizumab was a significant alternative to pembrolizumab based on the Chinese cost‐effectiveness threshold ($32,517.0/QALY) (Table 2).

**TABLE 2:** Summary of cost (US $) and outcome results in base‐case analysis

Strategy name	Mean cost ($)	Effect QALY (Mean)	Compared with	Incremental cost ($)	Incremental QALY	ICER ($)	Rank
Base cases							
Nivolizumab	226,164.9	0.96					1
Durvalumab	263,590.5	1.15	Nivolizumab	37,425.6	0.19	197,028.8	2
Pembrolizumab	268,273.0	1.34	Nivolizumab	42,108.1	0.38	111,859.0	4
			Durvalumab	4682.5	0.19	25,108.6	
Atezolizumab	265,923.7	1.49	Nivolizumab	39,758.9	0.52	76,182.3	3
			Durvalumab	2333.3	0.33	7029.2	
			Pembrolizumab	−2349.2	0.15	Dominated	
Price reduction							
Drug cost reduced by 20%							
Nivolizumab	215,686.4	0.96					1
Durvalumab	243,663.4	1.15	Nivolizumab	27,977.0	0.19	147,286.1	3
Pembrolizumab	245,955.5	1.34	Nivolizumab	30,269.2	0.38	80,409.2	4
			Durvalumab	2292.2	0.19	12,291.2	
Atezolizumab	242,730.3	1.49	Nivolizumab	27,043.9	0.52	51,819.0	2
			Durvalumab	−933.1	0.33	Dominated	
			Pembrolizumab	−3225.3	0.15	Dominated	
Drug cost reduced by 40%							
Nivolizumab	205,207.9	0.96					1
Durvalumab	223,736.3	1.15	Nivolizumab	18,528.4	0.19	97,543.4	4
Pembrolizumab	223,638.1	1.34	Nivolizumab	18,430.2	0.38	48,959.4	3
			Durvalumab	−98.1	0.19	Dominated	
Atezolizumab	219,536.8	1.49	Nivolizumab	14,328.9	0.52	27,455.7	2
			Durvalumab	−4199.5	0.33	Dominated	
			Pembrolizumab	−4101.3	0.15	Dominated	
Drug cost reduced by 60%							
Nivolizumab	194,729.4	0.96					1
Durvalumab	203,809.2	1.15	Nivolizumab	9079.7	0.19	47,800.7	4
Pembrolizumab	201,320.7	1.34	Nivolizumab	6591.3	0.38	17,509.6	3
			Durvalumab	−2488.5	0.19	Dominated	
Atezolizumab	196,343.3	1.49	Nivolizumab	1613.9	0.52	3092.4	2
			Durvalumab	−7465.9	0.33	Dominated	
			Pembrolizumab	−4977.4	0.15	Dominated	
Drug cost reduced by 80%							
Nivolizumab	184,250.9	0.96					4
Durvalumab	183,882.1	1.15	Nivolizumab	−368.9	0.19	Dominated	3
Pembrolizumab	179,003.3	1.34	Nivolizumab	−5247.7	0.38	Dominated	2
			Durvalumab	−4878.8	0.19	Dominated	
Atezolizumab	173,149.8	1.49	Nivolizumab	−11,101.1	0.52	Dominated	1
			Durvalumab	−10,732.2	0.33	Dominated	
			Pembrolizumab	−5853.5	0.15	Dominated	

Abbreviations: ICER, incremental cost‐effectiveness ratio; QALY, quality‐adjusted life years.

In Chinese health insurance, PD‐1/PD‐L1 inhibitors are the key items in insurance negotiations. The cost of these drugs will eventually reduce to 20% of the original cost. Therefore, to make accurate comparisons, the cost of each drug was reduced by 20%, 40%, 60%, and 80%. The results indicated that ICER gradually declined alongside costs. Other drugs such as atezolizumab and pembrolizumab might have advantages after cost reductions (Table 2).

### 3.3 Sensitivity analysis

**FIGURE 4:**
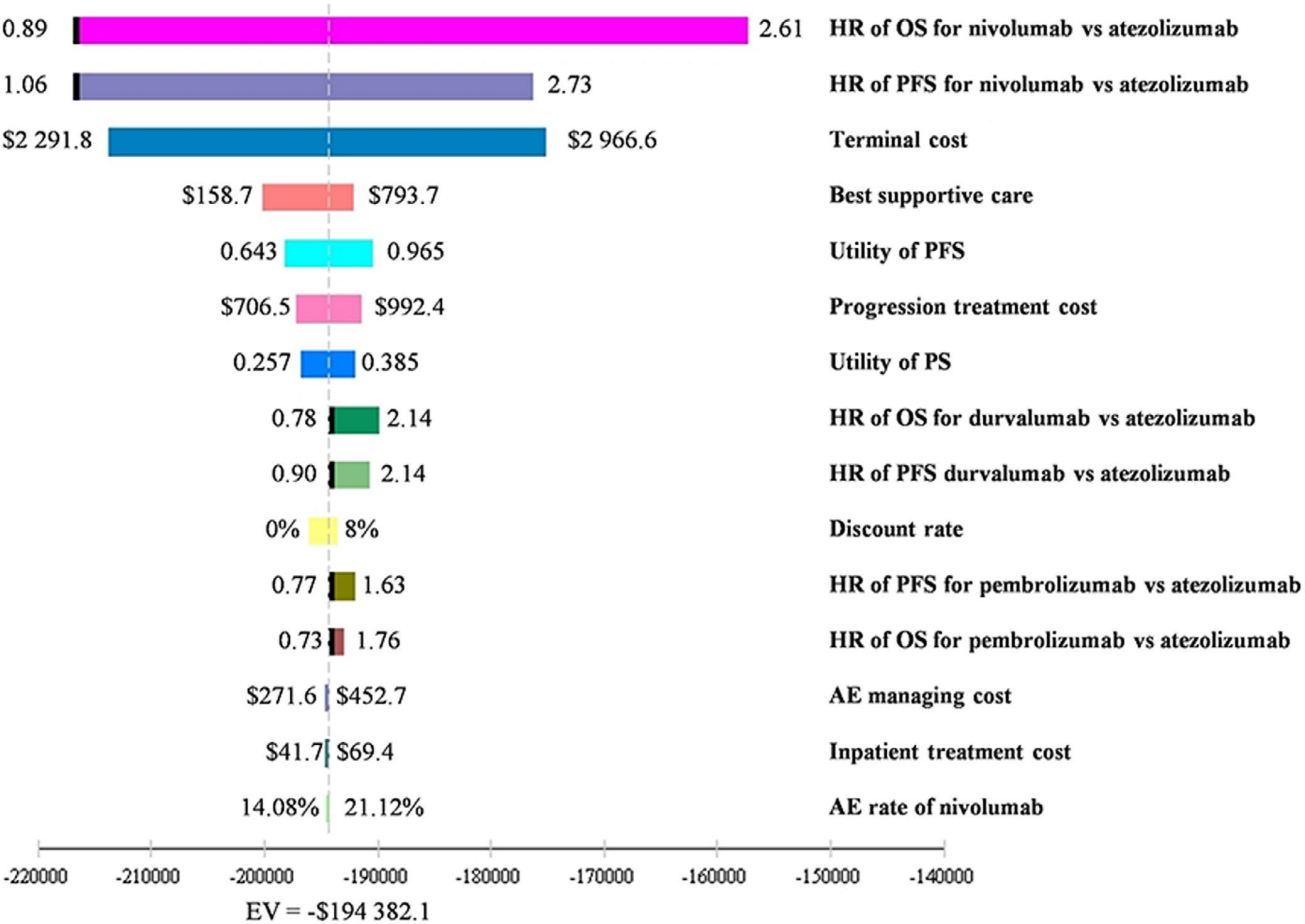
Tornado diagram of one‐way deterministic sensitivity analysis in China

**FIGURE 5:**
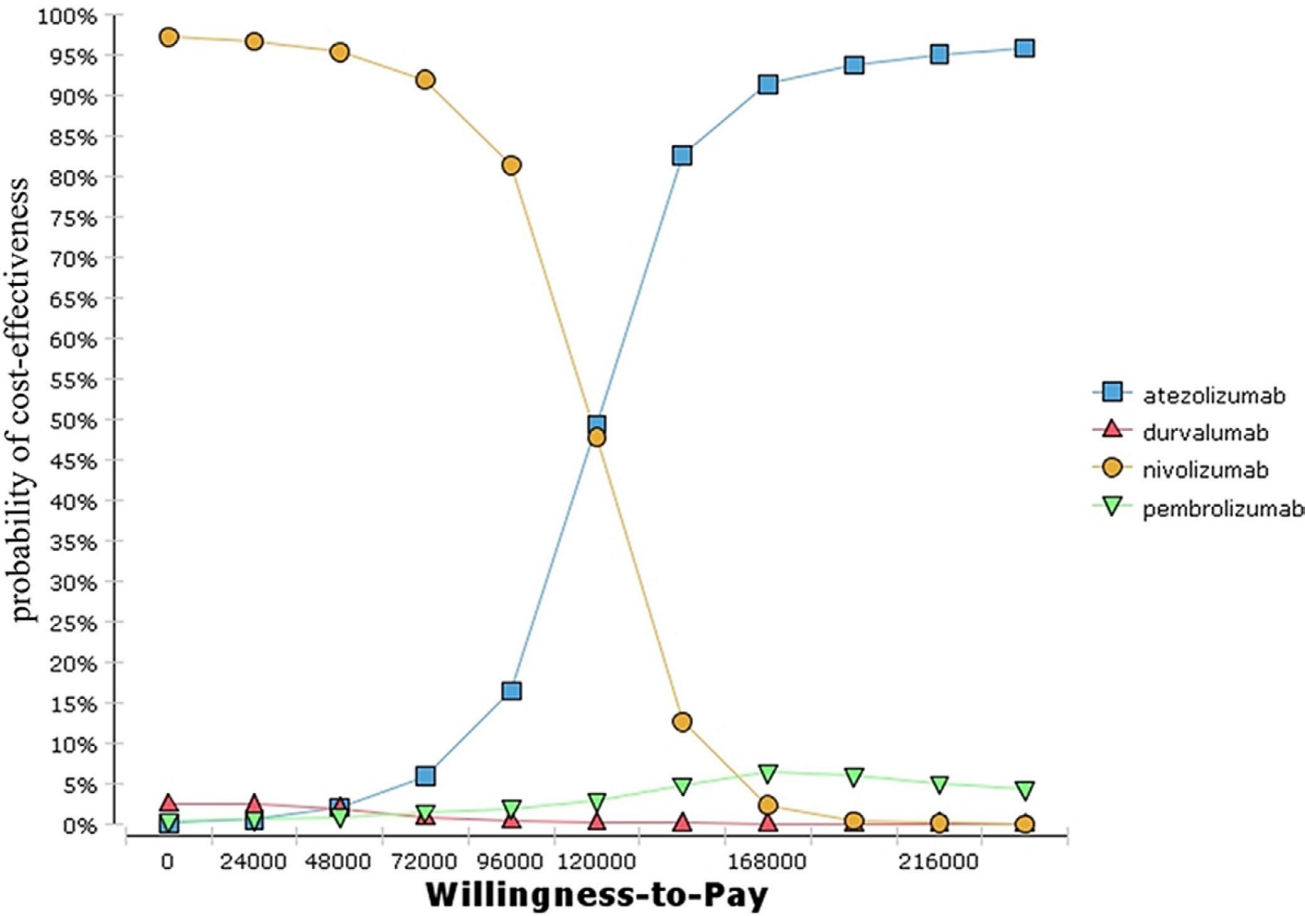
The cost‐effectiveness acceptability curves for base case analyses in China

One‐way sensitivity analyses indicated that the most influential parameters were the HRs of OS and PFS, utility, progression costs, and terminal treatment per cycle. However, adjusting these parameters might not yield substantial changes in ICER (Figure 4). The cost‐effectiveness acceptability curve indicated that nivolumab was the optimal treatment in 97.5% of the iterations with the Chinese willingness‐to‐pay threshold (Figure 5).

## 4 DISCUSSION

Cost‐effectiveness analysis is a key driver when resources are allocated to fund innovations. Immunotherapy appears to be promising as an effective treatment for NSCLC, but also appears as an expensive alternative to the current care standard.^21^ The PD‐1/PD‐L1 inhibitors clearly demonstrated an increase in life expectancy over the current chemotherapy standard, with an improved safety profile as a first‐line treatment.^12^, ^13^

NMAs were conducted to compare and benchmark the respective effectiveness and safety of pembrolizumab, nivolumab, atezolizumab, and durvalumab. The OS values of the results were similar among the four drugs, but pembrolizumab and atezolizumab had better PFS than nivolumab. Safety profiles indicated that atezolizumab had a higher risk of severe adverse events than pembrolizumab and nivolumab. However, effectiveness and safety were similar among the four drugs, which were not likely to optimize the dosage. Cost‐effectiveness was therefore appropriate for distinguishing between the drugs. The ICERs of the lifetime horizons of durvalumab, pembrolizumab, and atezolizumab against nivolumab were estimated at $ 197,028.8/QALY, $ 111,859.0/QALY, and $ 76,182.3/QALY, respectively. Other regimens, especially pembrolizumab and durvalumab, were strictly dominated by atezolizumab.

This study is the first that we are aware of that has examined the cost‐effectiveness of four competing, first‐line PD‐1/PD‐L1 inhibitors that are licensed and recommended in current clinical guidelines. Our results will be greatly significant for addressing resource limitations in healthcare settings. Recent economic evaluations raised a controversy that pembrolizumab is more cost‐effectiveness than chemotherapy in the first‐line treatment of advanced NSCLC with high‐level PD‐L1 expression.^22^, ^23^, ^24^ Compared with chemotherapy, pembrolizumab has been indicated as cost‐effectiveness for treating Swiss NSCLC patients, but unlikely to be cost‐effectiveness for Singaporean NSCLC patients. Moreover, nivolumab and atezolizumab also showed similar results in comparison with chemotherapy in cost‐effectiveness studies.^15^, ^25^, ^26^, ^27^ Although the efficacy of PD‐1/PD‐L1 inhibitors is significantly greater than that of chemotherapy, their cost is currently higher, and does not provide benefits for some patients. High drug costs have become the main driver for limiting widespread immunotherapy use for cancer and bring great burdens on both the patients themselves and society as a whole.^28^ In other words, the benefits of immunotherapy depend on economics in addition to effectiveness and safety. However, no previous studies have investigated the optimal PD‐1/PD‐L1 inhibitor for immunotherapy in NSCLC patients. Additionally, the survival status of cancer patients changes over time, and it is therefore very important for economic analyses to appropriately simulate the survival status.^29^, ^30^ The Kaplan–Meier survival curve can reflect the changes in the survival state over a period of time, but it is difficult to obtain the original data and evaluate the whole life cycle. Kaplan–Meier survival curves can reflect survival changes over time, but it is difficult to extract the original data and evaluate the entire life cycle. Therefore, according to current economic evaluations of chronic diseases such as cancer, the Markov model is often recommended to simulate changes in survival statuses during the life cycle.

This study is therefore derived from the real data of PFS and OS obtained from the IMpower 110 trial.^12^ Curve resimulation indicated that the model is better for clinical decision makers and management departments to refer to during relevant health decisions.

This study was subject to several limitations. First, due to the four investigated first‐line regimes having no previous evaluations within one trial, an NMA was conducted for an indirect comparison. Second, a Weibull survival model was used to simulate the lifetime outcomes. Third, some key clinical input data, such as data on the strategies for the PD‐1/PD‐L1 inhibitors, were extracted from clinical reality in China. Finally, the safety data did not include the grade 1 to 2 adverse events of PD‐1/PD‐L1 inhibitors that are not currently accepted treatments.

## 5 CONCLUSION

In summary, the efficacy and safety of the four PD‐1/PD‐L1 inhibitors analyzed in this study are similar. Only the OS was marginally better for pembrolizumab and atezolizumab than for nivolumab, and atezolizumab had significantly higher SAE safety outcomes than pembrolizumab and nivolumab. From the perspective of the Chinese healthcare system, nivolumab therapy is a cost‐effective alternative to other drugs as first‐line treatments for advanced NSCLC with high‐level PD‐L1 expression. Atezolizumab may be more cost‐effective than pembrolizumab and durvalumab as a first‐line treatment for locally advanced or metastatic NSCLC with high‐level PD‐L1 expression.

## DISCLAIMER

The views expressed are those of the authors. The funding agencies played no role in the study design, data collection and analysis, decision to publish, or manuscript preparation.

## PROVENANCE AND PEER REVIEW

Not commissioned; externally peer‐reviewed.

## CONFLICT OF INTEREST

The authors declare that there is no conflict of interest.

## ETHICS APPROVAL

This study was based on a literature review and modeling techniques; this study did not require approval by an institutional research ethics board.

## Supporting information

Supplementary Material

## References

[1] Global, regional, and national disability‐adjusted life‐years (DALYs) for 359 diseases and injuries and healthy life expectancy (HALE) for 195 countries and territories, 1990‐2017: a systematic analysis for the Global Burden of Disease Study 2017. Lancet 2018;392:1859‐1922. 30415748 10.1016/S0140-6736(18)32335-3 PMC6252083

[2] Wu B, Lu S. The effect of PD‐L1 categories‐directed pembrolizumab plus chemotherapy for newly diagnosed metastatic non‐small‐cell lung cancer: a cost‐effectiveness analysis. Transl Lung Cancer Res. 2020;9:1770‐1784. 33209600 10.21037/tlcr-19-605 PMC7653112

[3] Al‐Farsi A, Ellis PM. Treatment paradigms for patients with metastatic non‐small cell lung cancer, squamous lung cancer: first, second, and third‐line. Front Oncol. 2014;4:157. 25019058 10.3389/fonc.2014.00157 PMC4073307

[4] Thornton Snider J, Batt K, Wu Y, et al. The option value of innovative treatments for non‐small cell lung cancer and renal cell carcinoma. Am J Manag Care. 2017;23:e340‐e346. 29087638

[5] Li JX, Huang JM, Jiang ZB, et al. Current clinical progress of PD‐1/PD‐L1 immunotherapy and potential combination treatment in non‐small cell lung cancer. Integr Cancer Ther. 2019;18:1534735419890020. 31838881 10.1177/1534735419890020 PMC7242804

[6] Kumar R, Collins D, Dolly S, et al. Targeting the PD‐1/PD‐L1 axis in non‐small cell lung cancer. Curr Probl Cancer. 2017;41:111‐124. 28214087 10.1016/j.currproblcancer.2016.12.002

[7] Leal TA, Ramalingam SS. Immunotherapy in previously treated non‐small cell lung cancer (NSCLC). J Thorac Dis. 2018;10:S422‐s432. 29593888 10.21037/jtd.2018.01.141 PMC5861264

[8] Lu J, Ramirez RA. The role of checkpoint inhibition in non‐small cell lung cancer. Ochsner J. 2017;17:379‐387. 29230122 PMC5718450

[9] Carbone DP, Reck M, Paz‐Ares L, et al. First‐line nivolumab in stage IV or recurrent non‐small‐cell lung cancer. N Engl J Med. 2017;376:2415‐2426. 28636851 10.1056/NEJMoa1613493 PMC6487310

[10] Garon EB, Rizvi NA, Hui R, et al. Pembrolizumab for the treatment of non‐small‐cell lung cancer. N Engl J Med. 2015;372:2018‐2028. 25891174 10.1056/NEJMoa1501824

[11] Reck M, Rodríguez‐Abreu D, Robinson AG, et al. Pembrolizumab versus chemotherapy for PD‐L1‐positive non‐small‐cell lung cancer. N Engl J Med. 2016;375:1823‐1833. 27718847 10.1056/NEJMoa1606774

[12] Herbst RS, Giaccone G, de Marinis F, et al. Atezolizumab for first‐line treatment of PD‐L1‐selected patients with NSCLC. N Engl J Med. 2020;383:1328‐1339. 32997907 10.1056/NEJMoa1917346

[13] Rizvi NA, Cho BC, Reinmuth N, et al. Durvalumab with or without tremelimumab vs standard chemotherapy in first‐line treatment of metastatic non‐small cell lung cancer: the MYSTIC Phase 3 Randomized Clinical Trial. JAMA Oncol. 2020;6:661‐674. 32271377 10.1001/jamaoncol.2020.0237 PMC7146551

[14] Kitadai R, Okuma Y, Hakozaki T, et al. The efficacy of immune checkpoint inhibitors in advanced non‐small‐cell lung cancer with liver metastases. J Cancer Res Clin Oncol. 2020;146:777‐785. 31828427 10.1007/s00432-019-03104-w PMC11804625

[15] Giuliani J, Bonetti A. Immunotherapy in first‐line for advanced non‐small cell lung cancer: a cost‐effective choice? Recenti Prog Med. 2019;110:138‐143. 30968854 10.1701/3132.31141

[16] Chen R, Tao Y, Xu X, et al. The efficacy and safety of nivolumab, pembrolizumab, and atezolizumab in treatment of advanced non‐small cell lung cancer. Discov Med. 2018;26:155‐166. 30586539

[17] Almutairi AR, Alkhatib N, Martin J, et al. Comparative efficacy and safety of immunotherapies targeting the PD‐1/PD‐L1 pathway for previously treated advanced non‐small cell lung cancer: a Bayesian network meta‐analysis. Crit Rev Oncol Hematol. 2019;142:16‐25. 31326706 10.1016/j.critrevonc.2019.07.004

[18] Peters S, Reck M, Smit EF, et al. How to make the best use of immunotherapy as first‐line treatment of advanced/metastatic non‐small‐cell lung cancer. Ann Oncol. 2019;30:884‐896. 30912805 10.1093/annonc/mdz109

[19] Xiao J, Sun JF, Wang QQ, Qi X, Yao HY. Health economic evaluation reporting guideline and application status. Zhonghua Yu Fang Yi Xue Za Zhi. 2017;51:276‐280. 28260345 10.3760/cma.j.issn.0253-9624.2017.03.016

[20] Wu B, Li TE, Cai J, et al. Cost‐effectiveness analysis of adjuvant chemotherapies in patients presenting with gastric cancer after D2 gastrectomy. BMC Cancer. 2014;14:984. 25526802 10.1186/1471-2407-14-984 PMC4301844

[21] Zeng X, Karnon J, Wang S, et al. The cost of treating advanced non‐small cell lung cancer: estimates from the Chinese experience. PLoS One. 2012;7:e48323. 23118985 10.1371/journal.pone.0048323 PMC3485140

[22] Mok TSK, Wu Y‐L, Kudaba I, et al. Pembrolizumab versus chemotherapy for previously untreated, PD‐L1‐expressing, locally advanced or metastatic non‐small‐cell lung cancer (KEYNOTE‐042): a randomised, open‐label, controlled, phase 3 trial. Lancet. 2019;393:1819‐1830. 30955977 10.1016/S0140-6736(18)32409-7

[23] Nafees B, Lloyd AJ, Dewilde S, et al. Health state utilities in non‐small cell lung cancer: an international study. Asia Pac J Clin Oncol. 2017;13:e195‐e203. 26990789 10.1111/ajco.12477

[24] Gandhi L, Rodríguez‐Abreu D, Gadgeel S, et al. Pembrolizumab plus chemotherapy in metastatic non‐small‐cell lung cancer. N Engl J Med. 2018;378:2078‐2092. 29658856 10.1056/NEJMoa1801005

[25] Gubens MA, Davies M. NCCN guidelines updates: new immunotherapy strategies for improving outcomes in non‐small cell lung cancer. J Natl Compr Canc Netw. 2019;17:574‐578. 31117034 10.6004/jnccn.2019.5005

[26] Aziz MIA, Tan LE, Tan WHG, et al. Cost‐effectiveness analysis of pembrolizumab monotherapy versus chemotherapy for previously untreated advanced non‐small cell lung cancer. J Med Econ. 2020;23:952‐960. 32462958 10.1080/13696998.2020.1775620

[27] Zhou K, Jiang C, Li Q. Cost‐effectiveness analysis of pembrolizumab monotherapy and chemotherapy in the non‐small‐cell lung cancer with different PD‐L1 tumor proportion scores. Lung Cancer. 2019;136:98‐101. 31476529 10.1016/j.lungcan.2019.08.028

[28] Loong HH, Wong CKH, Leung LKS, et al. Cost effectiveness of PD‐L1‐based test‐and‐treat strategy with pembrolizumab as the first‐line treatment for metastatic NSCLC in Hong Kong. Pharmacoecon Open. 2020;4:235‐247. 31531842 10.1007/s41669-019-00178-7 PMC7248157

[29] Hu H, She L, Liao M, et al. Cost‐effectiveness analysis of nivolumab plus ipilimumab vs. chemotherapy as first‐line therapy in advanced non‐small cell lung cancer. Front Oncol. 2020;10:1649. 33014826 10.3389/fonc.2020.01649 PMC7507990

[30] Lin S, Luo S, Zhong L, et al. Cost‐effectiveness of atezolizumab plus chemotherapy for advanced non‐small‐cell lung cancer. Int J Clin Pharm. 2020;42:1175‐1183. 32524512 10.1007/s11096-020-01076-3

[31] Han J, Tian K, Yang J, et al. Durvalumab vs placebo consolidation therapy after chemoradiotherapy in stage III non‐small‐cell lung cancer: an updated PACIFIC trial‐based cost‐effectiveness analysis. Lung Cancer. 2020;146:42‐49. 32512272 10.1016/j.lungcan.2020.05.011

[32] Ding H, Xin W, Tong Y, et al. Cost effectiveness of immune checkpoint inhibitors for treatment of non‐small cell lung cancer: a systematic review. PLoS One. 2020;15:e0238536. 32877435 10.1371/journal.pone.0238536 PMC7467260

[33] Hoyle MW, Henley W. Improved curve fits to summary survival data: application to economic evaluation of health technologies. BMC Med Res Methodol. 2011;11:139. 21985358 10.1186/1471-2288-11-139 PMC3198983

[34] Djalalov S, Beca J, Ewara EM, et al. A comparison of different analysis methods for reconstructed survival data to inform cost‐effectiveness analysis. Pharmacoeconomics. 2019;37:1525‐1536. 31571137 10.1007/s40273-019-00830-4

