# Supplementary material for: Determining the optimal PD‐1/PD‐L1 inhibitors for the first‐line treatment of non‐small‐cell lung cancer with high‐level PD‐L1 expression in China
Source: Cancer Med. 2021 Aug 12;10(18):6344–53. doi: 10.1002/cam4.4191 (PMC8446572; doi:10.1002/cam4.4191)
Supplement: Supplementary file 1 — Supplementary Material [file CAM4-10-6344-s001.docx]

**Supplementary material**

Supplementary Table S1 The search criteria of this study

| #1 | (non-small-cell lung cancer) OR (non-small cell lung cancer) OR (non small-cell lung cancer) OR (non small cell lung cancer) OR (non-small-cell lung carcinoma) OR (non-small cell lung carcinoma) OR (non small-cell lung carcinoma) OR (non small cell lung carcinoma) OR (NSCLC) |
| --- | --- |
| #2 | (programmed death ligand 1) OR (PD-L1) OR (programmed death 1) OR (PD-1) OR (anti-programmed death ligand 1) OR (anti-PD-L1) OR (anti-programmed death 1) OR (anti-PD-1) OR (atezolizumab) OR (durvalumab) OR (nivolumab) OR (sintilimab) OR (toripalimab) OR (camrelizumab) OR (tislelizumab) OR (pembrolizumab) OR (avelumab) |
| #3 | (first-line) OR (first line) OR (treatment-naive) OR (treatmentnaïve) OR (untreated treatment) OR (therapy) |
| #4 | (randomized Controlled Trial) OR (controlled clinical trial) OR (randomized) OR (randomised) OR (randomly) OR (trial) OR (phase) |
| #5 | #1 AND #2 AND #3 AND #4 |

Supplementary Table S2 Basic information of included studies

| Trail | Reference Treatment | Comparator(s) | OS  HR (95%CI) | PFS  HR (95%CI) | AE(n/n) | SAE(n/n) |
| --- | --- | --- | --- | --- | --- | --- |
| KEYNOTE-042 | pembrolizumab  (n=636) | chemotherapy  (n=615) | 0.69  (0.56-0.85) | 0.81  (0.67,0.99) | 399/553 | 113/252 |
| KEYNOTE-024 | pembrolizumab  (n=154) | chemotherapy  (n=150) | 0.60  (0.41-0.89) | 0.50  (0.37-0.68) | 113/135 | 41/80 |
| CheckMate-026 | nivolumab  (n=267) | chemotherapy  (n=263) | 0.90  (0.63-1.29) | 1.07  (0.77-1.49) | 190/243 | 47/133 |
| IMpower110 | atezolumab  (n=286) | chemotherapy  (n=263) | 0.59  (0.40-0.89) | 0.63  (0.45-0.88) | 258/249 | 97/149 |
| MYSTIC | durvolumab  (n=369) | chemotherapy  (n=352) | 0.76  (0.55-1.04) | 0.87  (0.59-1.29) | 200/292 | 55/119 |

OS: overall survival; PFS: progression-free survival; AE: adverse events; SAE: severe adverse events; HR: hazard ratio; 95%CI: 95% confidence interval.


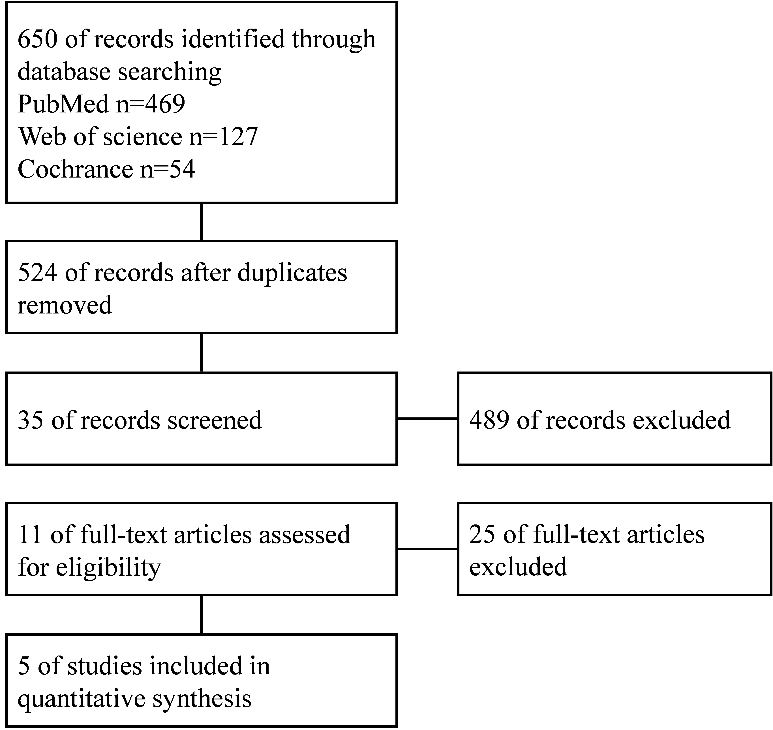


Supplementary Figure S1 Flow diagram of literature search and selection


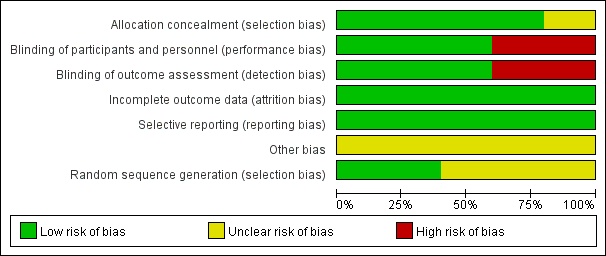


Supplementary Figure S2 the risk bias in treatment for non-small lung cancer.
